# Cucurbitacin B inhibits human breast cancer cell proliferation through disruption of microtubule polymerization and nucleophosmin/B23 translocation

**DOI:** 10.1186/1472-6882-12-185

**Published:** 2012-10-12

**Authors:** Suwit Duangmano, Phorntip Sae-lim, Apichart Suksamrarn, Frederick E Domann, Pimpicha Patmasiriwat

**Affiliations:** 1 School of Allied Health Science and Public Health, Walailak University, Bangkok, Thailand; 2 Department of Chemistry and Center of Excellence for Innovation in Chemistry, Faculty of Science, Ramkhamhaeng University, Bangkok, Thailand; 3 Free Radical and Radiation Biology Program, Department of Radiation Oncology, University of Iowa, Iowa City, IA, 52242, USA; 4 Faculty of Medical Technology, Mahidol University, Bangkok, Thailand

**Keywords:** Cucurbitacin B, Nucleophosmin/B23, Tubulin, Breast cancer

## Abstract

**Background:**

Cucurbitacin B, an oxygenated tetracyclic triterpenoid compound extracted from the Thai medicinal plant *Trichosanthes cucumerina* L*.*, has been reported to have several biological activities including anti-inflammatory, antimicrobial and anticancer. Cucurbitacin B is great of interest because of its biological activity. This agent inhibits growth of various types of human cancer cells lines.

**Methods:**

In this study, we explored the novel molecular response of cucurbitacin B in human breast cancer cells, MCF-7 and MDA-MB-231. The growth inhibitory effect of cucurbitacin B on breast cancer cells was assessed by MTT assay. The effects of cucurbitacin B on microtubules morphological structure and tubulin polymerization were analyzed using immunofluorescence technique and tubulin polymerization assay kit, respectively. Proteomic analysis was used to identify the target-specific proteins that involved in cucurbitacin B treatment. Some of the differentially expressed genes and protein products were validated by real-time RT-PCR and western blot analysis. Cell cycle distributions and apoptosis were investigated using flow cytometry.

**Results:**

Cucurbitacin B exhibited strong antiproliferative effects against breast cancer cells in a dose-dependent manner. We show that cucurbitacin B prominently alters the cytoskeletal network of breast cancer cells, inducing rapid morphologic changes and improper polymerization of the microtubule network. Moreover, the results of 2D-PAGE, real-time RT-PCR, and western blot analysis revealed that the expression of nucleophosmin/B23 and c-Myc decreased markedly after cucurbitacin B treatment. Immunofluorescence microscopy showed that cucurbitacin B induced translocation of nucleophosmin/B23 from the nucleolus to nucleoplasm. Treatment with cucurbitacin B resulted in cell cycle arrest at G_2_/M phase and the enhancement of apoptosis.

**Conclusions:**

Our findings suggest that cucurbitacin B may inhibit the proliferation of human breast cancer cells through disruption of the microtubule network and down-regulation of c-Myc and nucleophosmin/B23 as well as the perturbation in nucleophosmin/B23 trafficking from the nucleolus to nucleoplasm, resulting in G_2_/M arrest.

## Background

Cancer is the leading cause of death worldwide, accounting for 7.6 million deaths in 2008 [1]. The incident rate of cancer continues to increase largely due to the aging and growth of the world population and an increasing adoption of cancer-causing behaviors, particularly smoking, in developing countries [2]. Breast cancer is the most common cancer and the leading cause of cancer deaths among women in all races [3]. This cancer is usually treated with surgery and chemotherapy or/and radiation. However, undesired side effects may occur during breast cancer treatment [4,5]. Alternative medicine, such as the use of medicinal plants and derived natural products in cancer treatment, may reduce adverse side effects. The scientific evidence on the efficacy and safety still remains limited.

**Figure 1:**
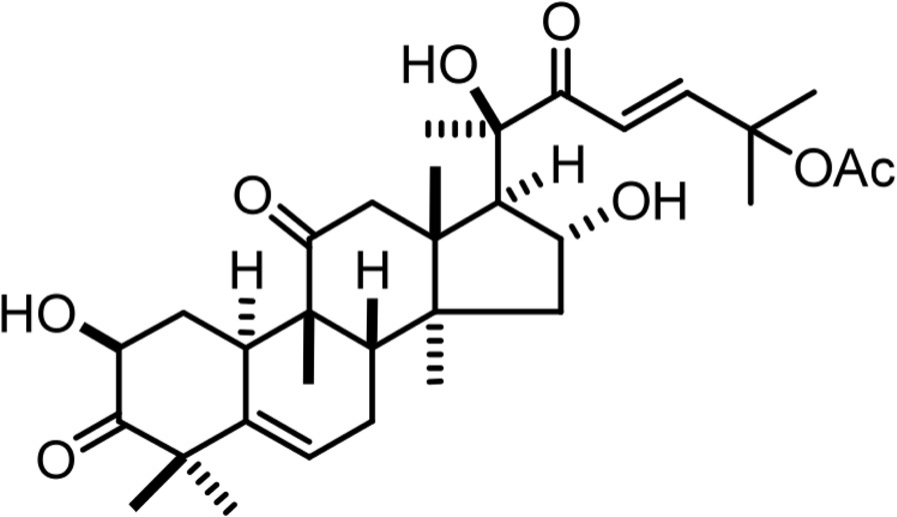
Chemical structure of cucurbitacin B (25-acetoxy-2β,16α,20β-trihydroxy-9β-methyl-19nor-10α-lanosta-5,23-diene-3,11,22-trione).

Cucurbitacins, oxygenated tetracyclic triterpenoid compounds extracted from cucurbitaceae plants, have been reported to have anti-inflammatory, antimicrobial and anticancer activities [6,7]. Cucurbitacins are classified into cucurbitacin A, B, C, D, E, F, I, L, 23, 24-dihydrocucurbitacin F, and hexanocucurbitacin F, as well as the three acetylated derivatives [8]. Among these compounds, cucurbitacin B is the most abundant form of cucurbitacins (Figure 1). Recently, it has been reported that cucurbitacin B inhibits growth of various types of human cancer cells and tumor xenografts. For instance, cucurbitacin B inhibits the proliferation of melanoma cells by inducing rapid depletion of the G-actin pool through ROS-dependent actin aggregation [9]. Moreover, cucurbitacin B inhibits both the STAT3 activation and the Raf/MEK/ERK pathway in K562 leukemia cell [10]. Chan et al. (2010) demonstrated that cucurbitacin B effectively restrains liver cancer xenograft through oral administration and this anticancer activity is contributable to the suppression of c-Raf and activation of ERK1/2 [11]. Previous studies also revealed that cucurbitacin B markedly inhibits growth of cancer cells and affects their cytoskeletal network [12,13]. Therefore, cucurbitacin B could probably affect the dynamics of microtubule stabilization.

Microtubule, a component of the cytoskeleton, is a dynamic heterodimer of α-tubulin and β-tubulin subunit. It plays crucial roles in various fundamental cell functions, including mitotic regulation, maintenance of cell morphology, intracellular motility and macromolecules and organelles trafficking [14,15]. Since microtubule is extremely essential for the process of mitosis, disruption of this structure can inhibit mitotic spindle regulation, arrest cell cycle progression at mitosis, and eventually lead to apoptosis [16,17]. There are two main types of microtubule-antimitotic targeted drugs used for treatment of highly proliferative malignant cells. Microtubule stabilizing drugs such as paclitaxel bind to β-tubulin and stabilize microtubules which could reduce their dynamicity, promote mitotic arrest and induce cell death [18,19]. Vincristine, a microtubule-destabilizing drug, binds to tubulin and inhibits microtubule formation, resulting in disruption of spindle assembly and arrest cell cycle at M phase [20,21].

In this work, we further explored the molecular mechanism response for cucurbitacin B isolated from Thai medicinal plant, *Trichosanthes cucumerina* L. in human breast cancer cells. Our data show that cucurbitacin B exhibits strong antiproliferative effects against breast cancer cells through disruption of microtubule polymerization and induces nucleophosmin/B23 translocation, causing cell cycle arrest at G_2_/M phase and induces apoptosis of breast cancer cells.

## Methods

### Cell lines and drug preparation

Human breast cancer cell lines (MDA-MB-231 (ER-, p53-), and MCF-7 (ER+, p53+)) were cultured in Dulbecco’s Modified Eagle’s Medium (DMEM/F12, Gibco, Grand Island, NY) supplemented with 10% fetal bovine serum (FBS, Gibco, Grand Island, NY), 100 U/mL penicillin and 100 μg/mL streptomycin (Gibco, Grand Island, NY) at 37°C in a humidified 5% CO_2_ incubator. Cells used in the study were in the exponential phase. Cucurbitacin B was obtained from the well known plant species, *Trichosanthes cucumerina* L. Briefly, the fruits of *C. cucumerina*, after ripening and became air-dry naturally, were collected from Nakhon Sawan province, Thailand. A voucher specimen of the plant and a dry fruit (Kimyong Chokepaiboon No. 002) are deposited at the Faculty of Science, Ramkhamhaeng University. The peels and seeds were removed to leave the fruit fibers, which were cut into small pieces and extracted successively with *n*-hexane and ethyl acetate. Column chromatography of the ethyl acetate extract gave, after recrystallization, pure cucurbitacin B as the major component Additional file 1. The spectroscopic (nuclear magnetic resonance and mass spectra) data were consistent with its structure and with the reported data [8]. This compound was dissolved in 10% dimethyl sulfoxide (DMSO) and diluted with DMEM/F12. Twenty-four hours after seeding, cucurbitacin B was added to the fresh culture medium to a various specified final concentration. Cells were incubated with cucurbitacin B for indicated times.

### MTT assay

The growth inhibitory effect of cucurbitacin B on breast cancer cells was assessed by the 3-(4,5-dimethylthiazol-2-yl)-2,5-diphenyltetrazolium bromide (MTT, Chemicon, Billerica, MA) assay. Cells (1×10^4^ cells/well) were seeded in 96-well plate and allowed to attach the well overnight. Cucurbitacin B was added to a final concentration (0.1, 1, 10, 100 μM) and the cells were incubated for further 48 hr. After incubation, 10 μl of 5 mg/mL MTT was added to the cells for 4 hr at 37°C. Afterthen, 100 μl isopropanol in 0.04 N HCl was added as the solubilizing agent. The absorbance at 570 nm was read using a microplate reader (Beckman coulter, Mississauga, ON) and the proportion of cell survival was calculated by dividing the average absorbance of the treated cells by the average of untreated cells. All experiments were performed in triplicate.

### Tubulin polymerization assay

The effect of cucurbitacin B on tubulin polymerization was analyzed using the Tubulin Polymerization Assay Kit (Cytoskeleton, Denver, CO). The assay was performed following the manufacturer’ guidance. Briefly, bovine brain tubulin protein was diluted with 420 μl of tubulin polymerization buffer containing 80 mM PIPES pH6.9, 2 mM MgCl_2_, 0.5 mM EGTA, 1 mM GTP, 10.2% glycerol to give a final concentration of 3 mg/mL tubulin. Then, the diluted tubulin was transferred to the pre-warm 96-well plate that contains 10 μl of cucurbitacin B (2.5, 5, and 10 μM), paclitaxel (10 μM), vincristine (10 μM), or vehicle (tubulin minus compound control). The polymerization of tubulin was measured at each consecutive minute during a period of 30 minutes at 37°C using the VarioSkan Flash microplate reader (Thermo, Helsinki, Finland) set at 340 nm.

### Cell cycle analysis

Cell cycle distribution was analyzed by flow cytometry. Cells (1x10^6^ cells/well) were treated with cucurbitacin B at various concentrations for 24 hr and harvested. The cells were trypsinized and resuspended in 1ml Dulbecco's Phosphate-Buffered Saline (DPBS). One million cells were centrifuged and suspended in 0.5 ml of Krishan reagent (0.1% Na citrate, 0.03% NP-40, 0.05 mg/mL Propidium iodide (PI), 0.02 mg/mL RNase A) before analysis. The stained cells were subjected to DNA content/cell cycle analysis using an LSR flow cytometer (BD Biosciences, Franklin Lakes, NJ) and analyzed using FlowJo software (Tree Star, Ashland, OR).

### Apoptosis analysis

The Annexin V–FITC Apoptosis Detection Kit (BD bioscience, Bedford, MA) was used to assess Annexin V-positive cells. Briefly, cells (1x10^6^ cells/well) were treated with cucurbitacin B at various concentrations for 24 hr and harvested. The freshly prepared cells were incubated with 1x Annexin binding buffer and Annexin V–FITC (2.5 μg/mL)-conjugated primary antibody for 15 min on ice. After incubation, 10 μg/mL PI was added to the suspension and the cells were measured by flow cytometry using an LSR flow cytometer (BD Biosciences, Franklin Lakes, NJ) and analyzed using FlowJo software (Tree Star, Ashland, OR).

### Proteomic analysis

Cells (5×10^5^ cells/well) were seeded into 6-well plates and treated with cucurbitacin B at various concentrations for 48 hr. Cell pellets were collected and lysed with 100 μl RIPA cell-lysis buffer (50 mM Tris pH 8.0, 150 mM NaCl, 0.1% SDS, 0.5% Na Deoxycholate, 1% TX-100) plus 1 mM NaF, 10 mM NaVO_4_, 10 mM PMSF, and 1/100 protease inhibitor cocktail (Sigma, St. Louis, MO). Protein extracts were separated according to their isoelcetric point (pI) followed by their molecular weight. Seven centimeter IPG strip (Bio-Rad, Hercules, CA) was rehydrated in 125 μl rehydration buffer containing 100μg protein samples for 16–24 hr at room temperature. Isoelectricfocusing was performed at 20°C with a limiting current 50 μA/IPG strip using the following parameter: 250 V for 20 min, 4,000 V for 2hr, 4,000 V for 10,000 V-hr. Focused gel was equilibrated for 10 min in 10 mL of Equilibration buffer I (6 M urea, 0.375 M Tris–HCl, pH 8.8, 2% SDS, 20% glycerol, 2% DTT) and 10 min in Equilibration buffer II (6 M urea, 0.375 M Tris–HCl, pH 8.8, 2% SDS, 20% glycerol, 2.5% iodoacetamide). For the second dimension, focused protein on IPG strip was separated on 12.5% polyacrylamide gel. Electrophoresis was performed at 20 mA/gel for the first 10 min following by 40 mA/gel. Gel was stained with Coomassie Brilliant Blue R-250 (Bio-Rad, Hercules, CA). The protein spots were excised from the stained gel and analyzed by LC-MS/MS mass spectrometer to obtain a mass peptide fingerprint. For identification of proteins, the peptide mass fingerprinting data were used to search in databases using Mascott program (Matrix Science, London, UK). The peptide mass fingerprinting of the proteins were scored with the Mowse score.

### Real-time RT-PCR

RNAs were extracted from the cells then the levels of mRNA of each gene (including c-Myc, STAT3, NUCLEOPHOSMIN/B23, and Tubulin) were determined by real-time RT-PCR. In brief, cells (5x10^5^ cells/well) were seeded into 6-well plate and treated with various concentration of cucurbitacin B for 48 hr. Total RNA was isolated from each cell line using the Qiagen RNeasy Mini Kit (Qiagen, Valencia, CA). Two micrograms of total RNA were reverse-transcribed with random primer according to the manufacture’s protocol using High-Capacity cDNA Reverse Transcription kit (Applied Biosystems, Foster City, CA). Real-time PCR was performed using Fast SYBR Green Master Mix (Applied Biosystems, Foster City, CA) with the Applied Biosystems 7500 Fast Real-Time PCR system (Applied Biosystems, Foster City, CA). The relative ratio of each gene was then calculated using the formula: 2^-ΔΔCt^ = 2^- {ΔCt(Cucurbitacin B-treated) - ΔCt(untreated)}^, where ΔCt=Ct(Genes)-Ct(GAPDH).

### Western blot analysis

Cells (1x10^6^ cells/well) were treated with various concentration of cucurbitacin B for 48 hr. The cell pellets were collected and lysed with 100 μl RIPA cell-lysis buffer (50 mM Tris pH 8.0, 150 mM NaCl, 0.1% SDS, 0.5% Na deoxycholate, 1% TX-100) plus 1 mM NaF, 10 mM NaVO_4_, 10 mM PMSF, and 1/100 protease inhibitor cocktail (Sigma, St. Louis, MO). Total protein was determined using Bio-Rad protein assay (Life science, Hercules, CA). Proteins were separated by 12.5% SDS-Polyacrylamide gels and electrotransferred onto nitrocellulose membranes before treating overnight with anti-nucleophosmin/B23, anti-STAT3, anti-tubulin, and anti-c-Myc (Santa Cruz Biotechnology, Santa Cruz, CA). Equal protein loading was confirmed on all immunoblots using GAPDH antibody (Santa Cruz Biotechnology, Santa Cruz, CA). Goat anti-rabbit IgG and goat anti-mouse IgG (BD Transduction Laboratories, San Diego, CA) were used as secondary antibodies against all primary antibodies. Protein bands were visualized by chemiluminescence with ECL plus reagent (Pierce, Rockford, IL) on a Typhoon FLA 7000 fluorescent detection system (GE Healthcare, Piscataway, NJ).

### Localization of nucleophosmin/B23

Cells (1x10^5^ cells/well) were seeded on cover slips in a 6-well plate overnight at 37°C. After removing the medium, the cells were treated with cucurbitacin B for 20 min and fixed with 4% formaldehyde in PBS for 30 min. Fixed cells were permeabilized with 0.5% Triton X-100 in PBS for 10 min, blocked in 2% BSA in PBS for 30 min, and washed three times in PBS. The cover slip was stained with anti-nucleophosmin/B23 antibody (Santa Cruz Biotechnology, Santa Cruz, CA) in blocking buffer for 1 hr, washed 3 times with PBS, and incubated with Alexa 568-conjugated anti-mouse IgG rabbit antibody (Molecular Probes, Eugene, OR) for an additional hour. DAPI dilution 1:1000 in PBS was used for nuclear staining (Sigma, St. Louis, MO). The cover slip was mounted with 50% glycerol in PBS on glass slide and sealed. The slide was visualized under Zeiss LSM 710 confocal microscopy (BD Biosciences, Franklin Lakes, NJ).

### Immunofluorescence detection of microtubules

The effect of cucurbitacin B on microtubules morphological structure was visualized by immunofluorescence microscopy. Cells (1x10^5^ cells/well) were seeded on cover slips in a 6-well plate overnight as mentioned above, treated with cucurbitacin B for 15 min and fixed with 4% formaldehyde in PBS for 30 min. Fixed cells were permeabilized, blocked, and washed as described above. Cover slip was stained with monoclonal anti-tubulin antibody dilution 1:500 in blocking buffer (Santa Cruz Biotechnology, Santa Cruz, CA) for 1 hr, washed 3 times with PBS and stained with 488 goat anti-rabbit IgG secondary antibody dilution 1: 5,000 in blocking buffer (Molecular Probes, Eugene, OR) for 1 hr. After washing with PBS, the cover slip was stained with DAPI dilution 1:1000 in PBS (Sigma, St. Louis, MO). The stained cover slip was mounted with 50% glycerol in PBS on glass slide and sealed. The slide was visualized under Zeiss LSM 710 confocal microscopy (BD Biosciences, Franklin Lakes, NJ).

### Statistic analysis

All experiments were performed in triplicate. Statistical analysis was analyzed using one-way ANOVA to compare the effect among control (without cucurbitacin B) and treated cells. *P* value < 0.05 was considered statistically significant.

## Results

### Cucurbitacin B exhibited antiproliferative activity against human breast cancer cells

**Figure 2:**
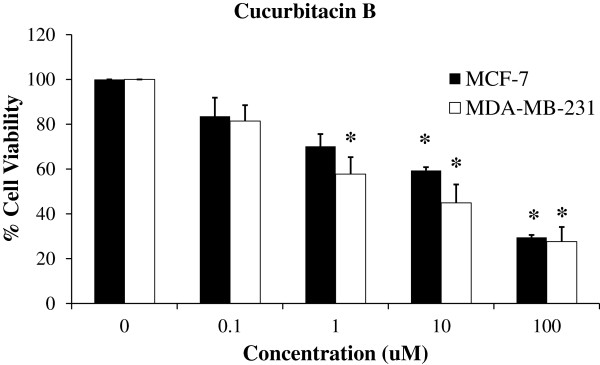
**Cucurbitacin B inhibits growth of human breast cancer cells.** MCF-7 and MDA-MB-231 were treated with cucurbitacin B at a final concentration, ranging from 0 to 100 μM for 48 hr. Growth inhibition was determined by the MTT assay. The percentage of cell survival was calculated by defining the absorption of cells without cucurbitacin B treatment as 100%. Results are the average of three independent experiments *, *p*<0.05 (treated *vs* untreated control).

To investigate the effect of cucurbitacin B on the proliferation of human breast cancer cells. MCF-7 and MDA-MB-231 cells were treated with the specified concentrations of cucurbitacin B for 48 hr. Cell viability was determined by MTT proliferation assay. The percentage of viability was calculated by defining the absorption of cells without cucurbitacin B treatment as 100%. Results are the average from three independent experiments. Cucurbitacin B extracted from the fruit fiber of *T. cucumerina* L. exhibited a dose-dependent inhibitory effect with the IC_50_ (mean inhibitory concentration that inhibited 50% growth) of 4.12 μM and 3.68 μM for MCF-7 and MDA-MB-231, respectively, as demonstrated in Figure 2. However, p53-mutant ER-/PR- and Her2- (triple-negative) MDA-MB-231 cell was more sensitive to cucurbitacin B than MCF-7.

### Cucurbitacin B caused cell cycle arrest at G_2_/M phase and induced apoptosis of breast cancer cells

**Figure 3:**
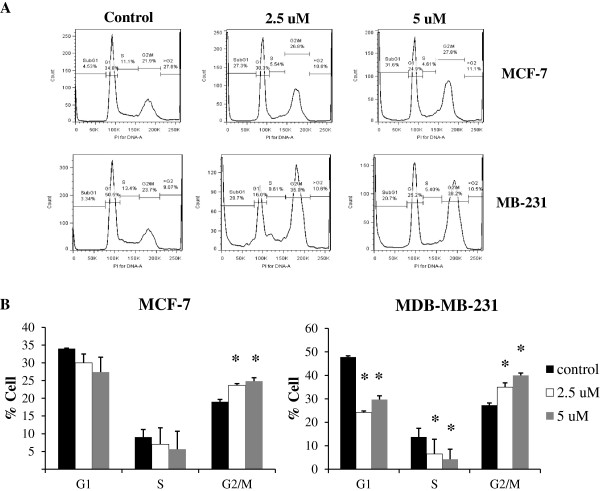
**Effect of cucurbitacin B on cell-cycle distribution.** MCF-7 and MDA-MB-231 were treated with 0, 2.5, and 5 μM cucurbitacin B for 24 hr, and then stained with propidium iodide (PI) before subjected to flow cytometric analysis. **A**. The cell cycle/DNA content histograms represent the cell population at each cell cycle phase as determined by the level of DNA content in each cucurbitacin B treatmented group. Blockage at G_2_/M and apoptotic induction was observed. **B**. The values indicate percentage of cells in each phases of the cell cycle. *, *p*<0.05 (treated *vs* untreated control).

**Figure 4:**
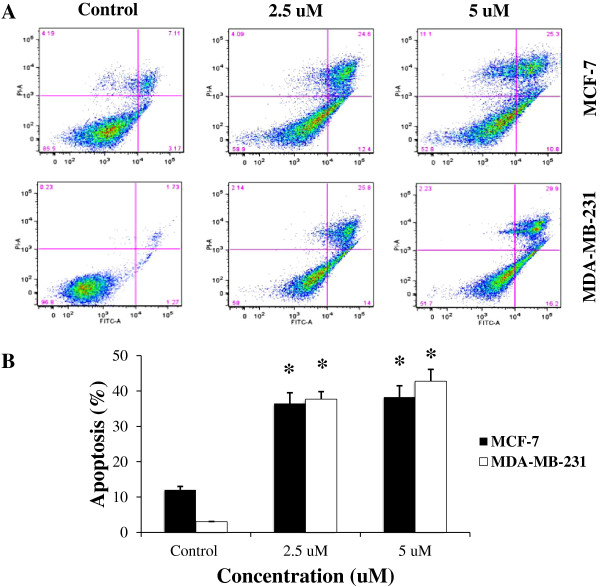
**Apoptotic induction by cucurbitacin B.** MCF-7 and MDA-MB-231 were incubated with cucurbitacin B for 24 hr and apoptosis was analyzed by Annexin V-FITC. **A**. Annexin V-FITC staining is represented on the X-axis and PI staining is represented on the Y-axis. **B**. The values indicate the percentage of apoptotic cells in each concentration. Results shown are the average of three independent experiments. *, *p*<0.05 (treated *vs* untreated control).

MCF-7 and MDA-MB-231 cells were treated with 2.5 μM and 5 μM cucurbitacin B for 24 hr, then stained with PI and subjected to flow cytometric analysis. The DNA histograms are representative of three independent experiments. Blockage at G_2_/M and apoptotic induction were observed in cucurbitacin B-treated cells. The treated cells were arrested at the G_2_/M phase of the cell cycle in both cell lines with decreased cell population in G1 and S phase (Figure 3). Moreover, the percentage of G_2_/M phase in cucurbitacin B-treated MDA-MB-231 cells is higher than in MCF-7 cells. The increase of cell in subG1 phase shown in the DNA histogram is the indicative of DNA fragmentation and apoptosis. Apoptosis was confirmed by staining the phosphatidylserine translocation with Annexin V-FITC. Results in Figure 4 indicated that cucurbitacin B treatment for 24 hr significantly induced apoptosis approximately 30% to 40% of breast cancer cells population.

### Determination of protein changes by cucurbitacin B treatment in breast cancer cells

The expression of target-specific proteins that are unique to the effect of cucurbitacin B in human breast cancer cells was determined by two-dimentional gel electrophoresis. Total proteins from untreated-cells and treated-cells were separated according to their isoelectric point (pI) and molecular weight (MW). Four separated peptide spots that showed significant changes in cucurbitacin B-treated cells compare to untreated cell were selected (Additional file 2). The proteins were identified by LC-MS/MS. As shown in Table 1, the result reveals that among the three proteins whose expressions were up-regulated, two of them, Hsp70 and β-subunit of prolyl 4-hydroxylase, belong to the heat shock protein class which could be up-regulated during stress conditions [22,23]. This might include the stress induced by cucurbitacin B. Of the four identified proteins, nucleophosmin/B23, an important nucleolar phosphoprotein was down-regulated. This protein functions in various cellular processes, such as ribosome biogenesis, centrosome duplication, cell cycle progression, apoptosis and cell differentiation [24].

**Table 1:** LC-MS/MS identification of the peptide spots in cucurbitacin B-treated cells

**Spot No.**	**NCBInr Entry**	**MW/pI**	**Protein identified**	**Expression**
CuB1	gi/825671	28/4.41	Nucleophosmin/B23	Down-regulated
CuB2	gi/31542947	61/5.59	Chaperonin	Up-regulated
CuB3	gi/20070125	57/4.61	Prolyl 4-hydroxylase	Up-regulated
CuB4	gi/16507237	72/4.92	Heat shock 70kDa	Up-regulated

Protein identification from 2-D gel.

**Figure 5:**
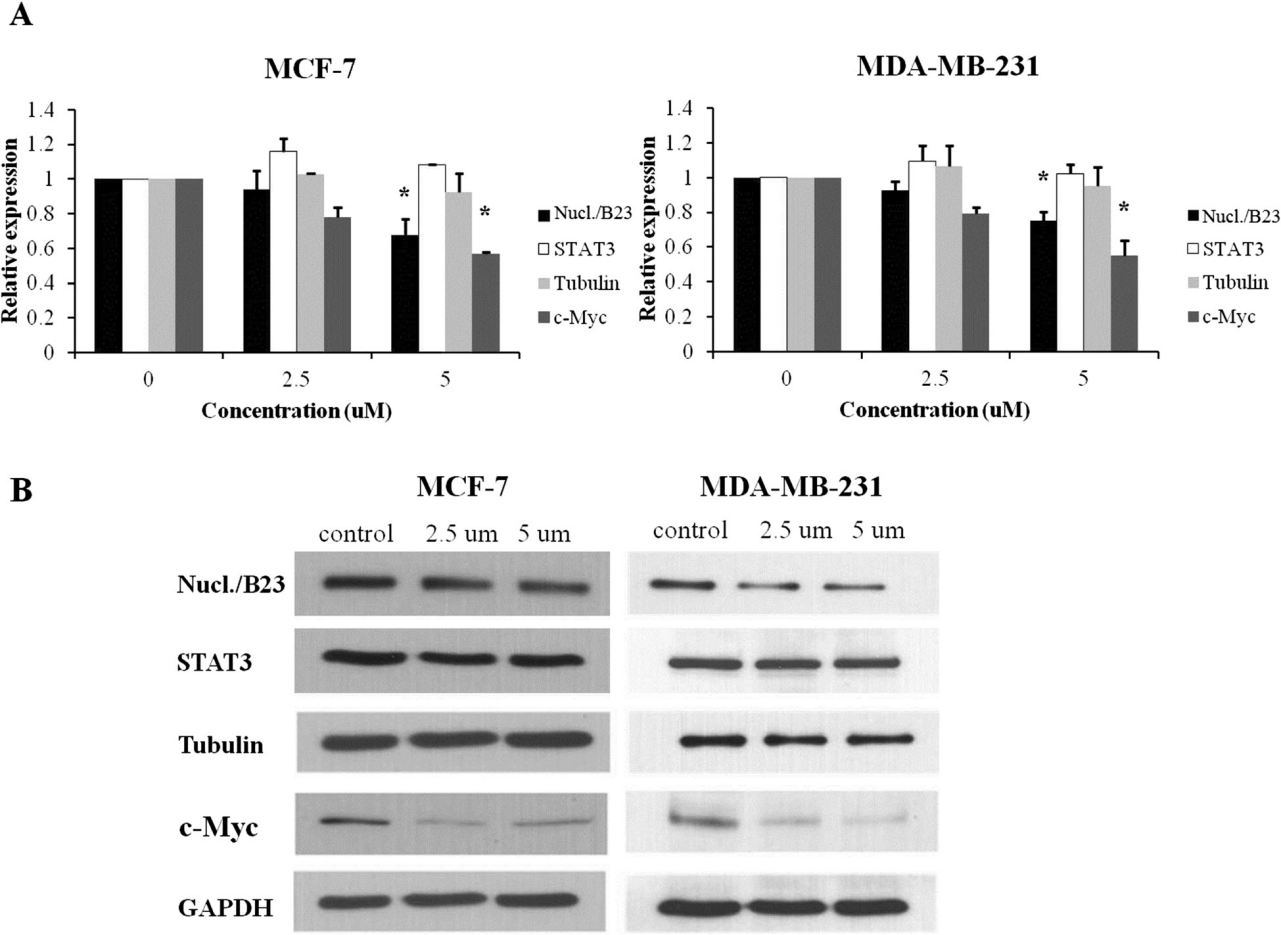
**Effect of cucurbitacin B on the level of nucleophosmin/B23, STAT3, tubulin, and c-Myc expression.** MCF-7 and MDA-MB-231 were treated with 0, 2.5, and 5 μM cucurbitacin B for 48 hr. **A**. RNA was extracted for real-time RT-PCR to quantitate the gene expression levels of nucleophosmin/B23, STAT3, tubulin, and c-Myc after cucurbitacin B treatment. Results shown are the average of three independent experiments. *, *P* < 0.05 (treated *vs* untreated control) **B**. Western blot was analyzed for protein expression. GAPDH was used as loading control.

To validate the identified proteins of interest, we performed real-time PCR and western blot analysis in order to determine the expression levels of these proteins in MCF-7 and MDA-MB-231 cells. Cells were incubated for 48 hr with the specified concentrations of cucurbitacin B and RNAs were extracted for real-time PCR to quantitate the expression levels of genes. After cucurbitacin B treatment, the expression of *nucleophosmin/B23* and *c-Myc* gene was decreased in dose-dependent manner but the decrease was not seen for STAT3 and tubulin in both cell types as shown in Figure 5A. The protein expressions of nucleophosmin/B23 and c-Myc were also decreased in dose-dependent manner but STAT3 and tubulin were not significantly different from the control in both cell lines, as demonstrated in Figure 5B.

### Cucurbitacin B induced nucleophosmin/B23 translocation from the nucleoli to the nucleoplasm

**Figure 6:**
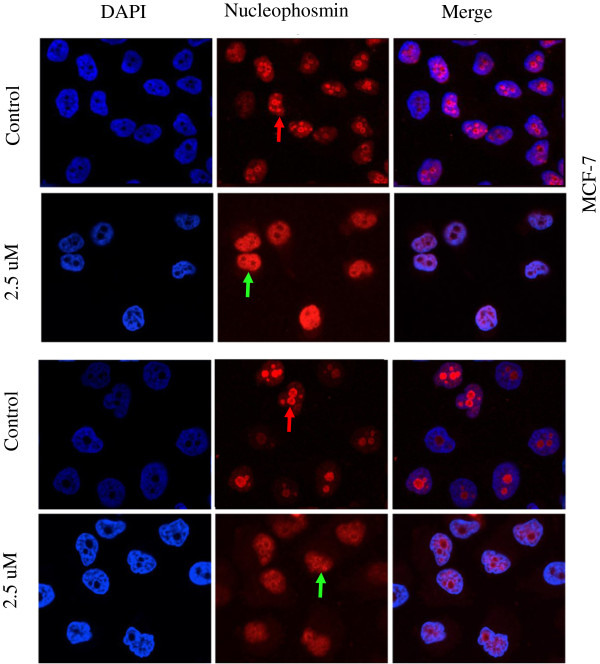
**Cucurbitacin B changes nucleophosmin/B23 localization in human breast cancer cells.** MCF-7 and MDA-MB-231 cells were stained with anti-nucleophosmin/B23 primary antibody. Alexa 568-conjugated anti-mouse IgG rabbit antibody was used as secondary antibody. DAPI was used for nuclear localization. Stained cells were analyzed by using confocal microscope. After exposure to 2.5 μM cucurbitacin B for 20 min. Translocation of nucleophosmin/B23 from nucleolus (red) to nucleoplasm (green) was observed.

To study the localization of nucleophosmin/B23 after cucurbitacin B treatment, cells were seeded on cover slips in six-well plate and treated with cucurbitacin B for 20 min. The cells were stained with anti-nucleophosmin/B23 antibody and analyzed with confocal microscopy. Figure 6 shows the cellular localization of nucleophosmin/B23 during cucurbitacin B treatment. Interestingly, nucleophosmin/B23-translocation was observed during cucurbitacin B treatment. The high intensity of nucleophosmin/B23 fluorescence was observed mainly within the nucleoli region of the untreated control cells and the intensity was weak in nuclear matrix region. After cucurbitacin B treatment, the distribution of nucleophosmin/B23 was significantly altered. The fluorescent intensity within the nucleolus was obviously decreased, while the strong fluorescence was observed in nuclear matrix outside nucleolus. Hence, cucurbitacin B induced nucleophosmin/B23 translocation from nucleolus to nucleoplasm.

### Cucurbitacin B caused aggregation of α-tubulin

**Figure 7:**
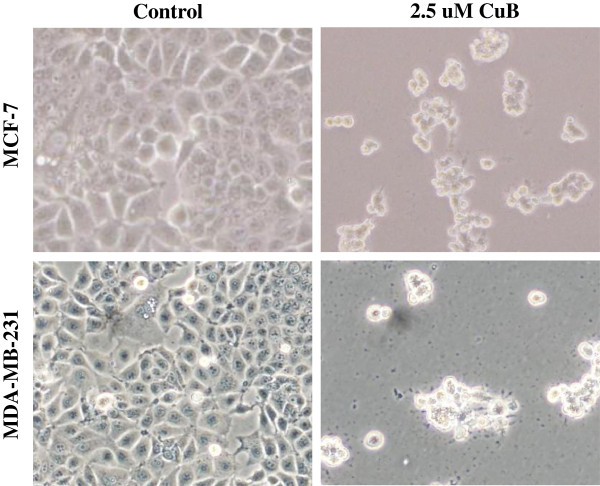
**Morphological changes by cucurbitacin B treatment of breast cancer cells.** MCF-7 and MDA-MB-231 cells were treated with 2.5 μM cucurbitacin B for 24 hr, and the cells were visualized under phase-contrast microscopy to investigate the morphological alteration. Shrinkage and rounding of the cell shape were observed in both cell types.

**Figure 8:**
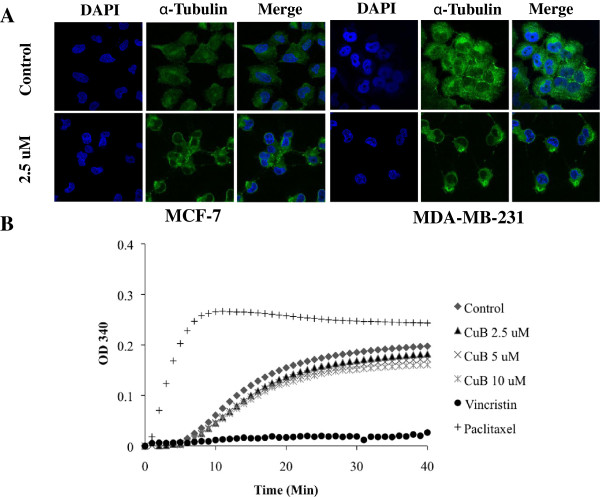
**Cucurbitacin B inhibits microtubule polymerization. A**. Effect of cucurbitacin B on the organizations of cellular microtubule network. Immunofluorescent staining for α-tubulin was inspected in MCF-7 and MDA-MB-231 cells treated with cucurbitacin B. Cells were fixed, permeabilized and stained with anti-α-tubulin monoclonal antibody. The cells were analyzed using confocal microscopy. DAPI was used for nuclear localization. Change of tubulin organization within the cells was observed after cucurbitacin B treatment. **B**. Effect of cucurbitacin B on microtubule polymerization in a cell-free system was determined in vitro. Bovine brain tubulin protein (3 mg/mL) was mixed with special reaction buffer as described in materials and method, and incubated with 2.5, 5, 10 μM cucurbitacin B, 10 μM vincristine or 10 μM paclitaxel. The polymerization of tubulin was determined on the basis of the increase in optical density. The O.D. (340 nm) was measured each minute for up to 40 minutes at 37°C using microplate reader. Unlike paclitaxel and vincristine, treatment with cucurbitacin B in cell-free system has no effect on tubulin density.

Treatment of MCF-7 and MDA-MB-231 cells with cucurbitacin B showed the rapid and dramatic morphological changes including cell shrinkage and rounding of the cell shape under light microscope speculation (Figure 7), pointing to the possibility of microtubule network disruption. To study the effect of cucurbitacin B on the structure organization of cellular microtubules, MCF-7 and MDA-MB-231 cells were seeded on cover slips in six-well plate and treated with cucurbitacin B for 15 min. Cells were stained with anti-α-tubulin antibody. DAPI was used for nuclear staining and then analyzed by using confocal microscope. Figure 8A shows the microtubules network in untreated control cells which display intact organization and arrangement. Compared with the control cells, cucurbitacin B treated cells exhibited obvious aggregation of α-tubulin. The changes were observed within 15 min during incubation with cucurbitacin B.

We further determined whether cucurbitacin B could inhibit microtubule assembly in vitro using tubulin polymerization assay kit in cell-free system. Bovine brain tubulin was mixed with 2.5 μM, 5 μM, and 10 μM cucurbitacin B and then monitored the effect of cucurbitacin B on polymerization of tubulin at 340 nm microplate reader as described. The result in Figure 8B shows that cucurbitacin B did not disrupt the polymerization of bovine tubulin even in the maximum concentration of cucurbitacin B, suggesting that cucurbitacin B does not act directly on microtubule assembly, which is different from the effects of paclitaxel and vincristine, antimicrotubules drugs.

## Discussion

*Trichosanthes cucumerina* L*.* (Cucurbitaceae family), commonly found in Southeast Asia and Australia, is traditionally used for the treatment of helmintic, diabetic and inflammatory diseases in Thailand. Previous study reported that both root extract and fruit juice of *T. cucumerina* have cytotoxic effect against various types of human cancer cells [25]. Cucurbitacin B, one of the most abundant forms of cucurbitacins extracted from the fruit of *T. cucumerina*, is known for its strong anticancer activity. Our previous studies have shown that cucurbitacin B exerts anticancer effect by inhibiting telomerase via down regulation of both the hTERT and c-Myc [26]. The cell cycle arrest at G_2_/M phase and apoptotic induction through the reduction of Wnt associated proteins by cucurbitacin B were shown. In addition, reduced translocation of galactin-3-mediated β-catenin to the nucleus in breast cancer cells by this agent has been reported [27]. Noteworthy, cucurbitacin B showed only a slight effect on the proliferation of a non-malignant HBL-100 cells [26,27]. We further examined the alternative mechanism of cucurbitacin B on human breast cancer cells inhibition in aggressive cancer cell types in which the treatment is problematic. The MDA-MB-231 was chosen for study since this cell type exhibits highly aggressive, triple negative characteristic.

Recently, the effects of cucurbitacin B on cytoskeletal network have been reported. Haritunians et al. (2008) showed that cucurbitacin B prominently alters the cytoskeletal network of leukemic cells by induces improper polymerization and subsequent aggregation of F-actin [28]. Treatment of human glioblastoma multiforme (GBM) cells with cucurbitacin B significantly changed their morphology, causing F-actin to be clumped. The cells were then rounded and refractiled and the microtubule assembly was disorganized [12]. Similarly, we show that exposure to cucurbitacin B could cause shrinkage and rounding of the cell shape (Figure 7). These morphological changes are hallmark features of apoptosis, indicating that cucurbitacin B induces apoptosis in human breast cancer cells. Our result also indicates that cucurbitacin B is able to induce cell arrest at G_2_/M phase of the cell cycle and finally triggers apoptosis in the two breast cancer cells tested (Figure 3 and Figure 4).

Since the formation of spindle fibers for chromosome separation during mitosis is critical to the G_2_/M transition process, disruption of spindle function by drug-induced suppression of microtubule dynamics could block cell cycle progression at the G_2_/M phase [14]. As indicated by immunoflourescent staining (Figure 8A), disruption of the microtubule network after cucurbitacin B treatment was observed herein. Cucurbitacin B-treated cells became more spherical and exhibited aggregation of α-tubulin. Normal filamentous organization of microtubules were scarcely observed after treatment with 2.5 μM of cucurbitacin B. This finding suggests that cucurbitacin B disrupts tubulin polymerization to microtubule in the cells. However, our data reveals that cucurbitacin B might not bind directly to tubulin or microtubules. Adding paclitaxel into the reaction clearly demonstrated the increase in tubulin polymerization, whereas adding vincristine provided the opposite result as the drug decrease tubulin polymeric intensity. Surprisingly, in this cell-free system cucurbitacin B solely did not cause any changes on tubulin polymerization, suggesting that some additional factors within cells could be involved in tubulin polymerization.

There are many cellular regulatory factors that can affect the polymerization of microtubules. These include microtubule-associated proteins (MAPs) such as dynein and kinesin motor proteins, and microtubule-regulatory proteins such as survivin, stathmin, and dynactin [15,17,29]. Natural and synthetic compounds that target microtubule-associated proteins are among the most successful and widely used cancer chemotherapeutic agents. For example, a new natural product extracted from a soil fungus named terpendole E, has shown to target human kinesin Eg5 leading to mitotic arrest [30]. Cucurbitacin B might also indirectly disrupt the polymerization of microtubules via affecting some regulatory proteins that function on microtubule assembly. However, the precise cooperative mediator of cucurbitacin B for interfering the tubulin polymerization is unclear and further study is needed.

To identify the target molecules of cucurbitacin B in breast cancer cells, we additionally explored proteomic analysis. Results of 2D-PAGE, real-time RT-PCR, and western blot analysis revealed that the expression of nucleophosmin/B23 and c-Myc decreased markedly after cucurbitacin B treatment. We show that cucurbitacin B induced the translocation of nucleophosmin/B23 from the nucleolus to nucleoplasm (Figure 6). Nucleophosmin/B23 distributes mostly in the nucleolus of the untreated cells. It is a multifunctional phosphoprotein shuttling continuously between nucleolus and cytoplasm. This protein has an important role in controlling cellular cycling activities related to both cell proliferation and apoptosis [31]. Previous studies showed that nucleophosmin/B23 is down-regulated in cancer cells during drug-induced apoptosis [32,33]. There are several anticancer drugs (i.e., actinomycin D, daunomycin, camptothecin) known to induce translocation of nucleophosmin/B23 from nucleoli to the nucleoplasm and triggers apoptosis [34-36]. Thus, the changes of localization and expression of nucleophosmin/B23 could play crucial role in apoptotic process. Our finding that cucurbitacin B could induce nucleophosmin/B23 translocation in the same way as the other anticancer drugs mentioned above suggests that cucurbitacin B can induce apoptosis in breast cancer cells. Moreover, nucleophosmin/B23 was reported to inhibit Eg5-mediated microtubule depolymerization. Cells lacking nucleophosmin/B23 exhibited a disrupted microtubule network with a lower level of polymerized tubulin. Therefore, nucleophosmin/B23 plays a protective role in microtubule polymerization. Cucurbitacin B could down-regulate the expression of nucleophosmin/B23, leading to disrupt the polymerization of mitotic spindle, arrest the cell cycle at G_2_/M phase and induce apoptosis.

The c-Myc has been proposed to be involved in multiple cellular functions including cell cycle regulation, differentiation and apoptosis [37,38]. In HL60 cells, c-Myc interacts with polymerized microtubules. This polymerized protein acts as a reservoir to sequester the c-Myc protein [39]. Pretreatment by tubulin inhibitory agents either stabilizing or depolymerizing tubulins. This drugs decrease c-Myc expression in human colon carcinoma cell HT29-D4 [40]. Thus, cucurbitacin B may decrease the expression of c-Myc via disruption of tubulin polymerization. c-Myc is able to bind promoter of nucleophosmin/B23 at Myc-binding site and regulates the expression of nucleophosmin/B23 [41]. Recently, retinoic acid (RA) has been reported to reduce the expression of c-Myc and also affect the binding of c-Myc to nucleophosmin/B23 expression, leading to down-regulated expression of nucleophosmin/B23 [42]. Thus, c-Myc is one of the factors that regulate nucleophosmin/B23 promoter. In this study, we found that the expressions of both nucleophosmin/B23 and c-Myc were decreased after cucurbitacin B treatment. Therefore, down-regulation of nucleophosmin/B23 during cucurbitacin B treatment may be a consequence of the decreased expression of c-Myc and affected binding of c-Myc to nucleophosmin/B23 promoter. Taken together, cucurbitacin B disrupts the polymerization of microtubules, induces trafficking of nucleophosmin/B23 from nucleolus to nucleoplasm as well as down-regulates the expression of c-Myc and nucleophosmin/B23.

## Conclusions

In conclusion, we have elucidated the novel molecular responses for cucurbitacin B treatment. Cucurbitacin B inhibits breast cancer cells proliferation through disruption of microtubule polymerization and induced nucleophosmin/B23 translocation, causing cell cycle arrest at G_2_/M phase and induced apoptosis. Therefore, cucurbitacin B could be a potentially useful as a leading agent for further anti-breast cancer research, as well as for in vivo and clinical studies aimed for breast cancer therapy.

## Abbreviations

ROS: Reactive oxygen species; DMEM: Dulbecco’s Modified Eagle Medium; FBS: Fetal bovine serum; DMSO: Dimethylsulfoxide; MTT: 3-(4, 5-dimethylthiazol-2-yl)-2,5-diphynyl tetrazolium bromide; PI: Propidium Iodide; DAPI: 4,6-diamidino-2-phenylindole; PBS: Phosphate buffer saline; ER: Estrogen receptor; FITC: Fluorescein isothiocyanate; LC-MS/MS: Liquid chromatography-Mass spectrometry; MAP: Microtubule associated protein; RA: Retinoic acid.

## Competing interests

The authors declare that there are no conflicts of interest regarding the contents of this article.

## Authors’ contributions

SD: Project design, experimental works, data analysis, generated the figures and manuscript preparation. PS and AS: Cucurbitacin B provider, participating in extraction, isolation and purification. FD: Project design, data analysis and project coordination. PP: Project design, conceptual planning, project coordination and manuscript preparation. All authors have read and approved of the final version of the manuscript.

## Pre-publication history

The pre-publication history for this paper can be accessed here:

http://www.biomedcentral.com/1472-6882/12/185/prepub

## Supplementary Material

Additional file 1 **Extraction and isolation of cucurbitacin B. ** The dried fruit fibers of * T. cucumerina * L. (2.72 kg) were chilled in liquid N_2_, milled to small pieces and extracted successively with *n*-hexane, EtOAc and MeOH in a Soxhlet extraction apparatus. The extracts were evaporated to dryness under reduced pressure at temperature 40-45°C. The hexane extract (greenish viscous oil, 14.9 g), the EtOAc extract (greenish sticky solid, 83.2 g) and the MeOH extract (dark brownish amorphous, 257.8 g) were respectively obtained. The extraction sequence is shown in Figure 1. Click here for file

Additional file 2 **Figure Protein expression of nucleophosmin/B23 (Nucl./B23), STAT3, tubulin, and c-Myc were determined by western blot analysis.** MCF-7 and MDA-MB-231 were treated without or with cucurbitacin B for 48 hrs. After incubation, total proteins were extracted and performed western blotting to analyze the expression levels of nucleophosmin, STAT3, tubulin, and c-Myc gene. This bar graph represents the densitometric analyses of expression of nucleophosmin/B23 (Nucl./B23), STAT3, tubulin, and c-Myc relative to the untreated control. * *P* < 0.05 (treated *vs* untreated control). Click here for file
