# Supplementary material for: Cucurbitacin B inhibits human breast cancer cell proliferation through disruption of microtubule polymerization and nucleophosmin/B23 translocation
Source: BMC Complement Altern Med. 2012 Oct 12;12:185. doi: 10.1186/1472-6882-12-185 (PMC3527297; doi:10.1186/1472-6882-12-185)
Supplement: Additional file 1 — Extraction and isolation of cucurbitacin B. The dried fruit fibers of T. cucumerina L. (2.72 kg) were chilled in liquid N2, milled to small pieces and extracted successively with n-hexane, EtOAc and MeOH in a Soxhlet extraction apparatus. The extracts were evaporated to dryness under reduced pressure at temperature 40-45°C. The hexane extract (greenish viscous oil, 14.9 g), the EtOAc extract (greenish sticky solid, 83.2 g) and the MeOH extract (dark brownish amorphous, 257.8 g) were respectively obtained. The extraction sequence is shown in Figure 1. [file 1472-6882-12-185-S1.doc]

**Extraction and isolation of cucurbitacin B**

The dried fruit fibers of *T.* *cucumerina* L. (2.72 kg) were chilled in liquid N_2_, milled to small pieces and extracted successively with *n*-hexane, EtOAc and MeOH in a Soxhlet extraction apparatus. The extracts were evaporated to dryness under reduced pressure at temperature 40-45 ^o^C. The hexane extract (greenish viscous oil, 14.9 g), the EtOAc extract (greenish sticky solid, 83.2 g) and the MeOH extract (dark brownish amorphous, 257.8 g) were respectively obtained. The extraction sequence is shown in Figure 1

Dried fruit fiber of *T.* *cucumerina* (2.72 kg)

1. Soxhlet extraction with *n*-hexane
2. Solution filtered

Hexane evaporated

Hexane extract

(14.9 g)

Marc

1. Soxhlet extraction with EtOAc
2. Solution filtered

EtOAc evaporated

Marc

EtOAc extract

(83.2 g)

1. Soxhlet extraction

with EtOAc

1. Solution filtered

MeOH evaporated

Marc

MeOH extract

(257.8 g)

**Figure 1** Extraction of the dried fruit fiber of *T*. *cucumerina* L*.*

The EtOAc extract (83.2 g) was fractionated by quick column chromatography (QCC) (Merck silica gel 60 PF _254_, 500 g) then, eluting with CH_2_Cl_2_, CH_2_Cl_2_-MeOH and MeOH with increasing amount of the more polar solvent. The eluates were examined by TLC, 8 groups of eluting fractions were obtained and to give cucurbitacin B (9.10 g) (Figure 2), which gave purple coloration with the anisaldehyde reagent.

EtOAc extract (83.2 g)

QCC

CH_2_Cl_2_, CH_2_Cl_2_-MeOH and MeOH

Gr.2

4-12

Gr.4

18-47

Gr.6

74-89

Gr.8

153-186

Gr.3

13-17

Gr.5

48-73

Gr.7

90-152

Gr.1

1-3

Compound 16

Cucurbitacin B

Gr. 1, Fraction 1-3 (19.22 g) Gr. 2, Fraction 4-12 (4.48 g)

Gr. 3, Fraction 13-17 (3.15 g) Gr. 4, Fraction 18-47 (8.23 g)

Gr. 5, Fraction 48-73 (7.05 g) Gr. 6, Fraction 74-89 (7.03 g)

Gr. 7, Fraction 90-152 (9.10 g) Gr. 8, Fraction 153-186 (2.40 g)

**Figure 2** Fractionation of the EtOAc extract of *T. cucumerina* L*.*
