# Supplementary material for: Cucurbitacin B inhibits human breast cancer cell proliferation through disruption of microtubule polymerization and nucleophosmin/B23 translocation
Source: BMC Complement Altern Med. 2012 Oct 12;12:185. doi: 10.1186/1472-6882-12-185 (PMC3527297; doi:10.1186/1472-6882-12-185)
Supplement: Additional file 2 — Figure Protein expression of nucleophosmin/B23 (Nucl./B23), STAT3, tubulin, and c-Myc were determined by western blot analysis. MCF-7 and MDA-MB-231 were treated without or with cucurbitacin B for 48 hrs. After incubation, total proteins were extracted and performed western blotting to analyze the expression levels of nucleophosmin, STAT3, tubulin, and c-Myc gene. This bar graph represents the densitometric analyses of expression of nucleophosmin/B23 (Nucl./B23), STAT3, tubulin, and c-Myc relative to the untreated control. * P < 0.05 (treated vs untreated control). [file 1472-6882-12-185-S2.doc]

**Supplemental data**


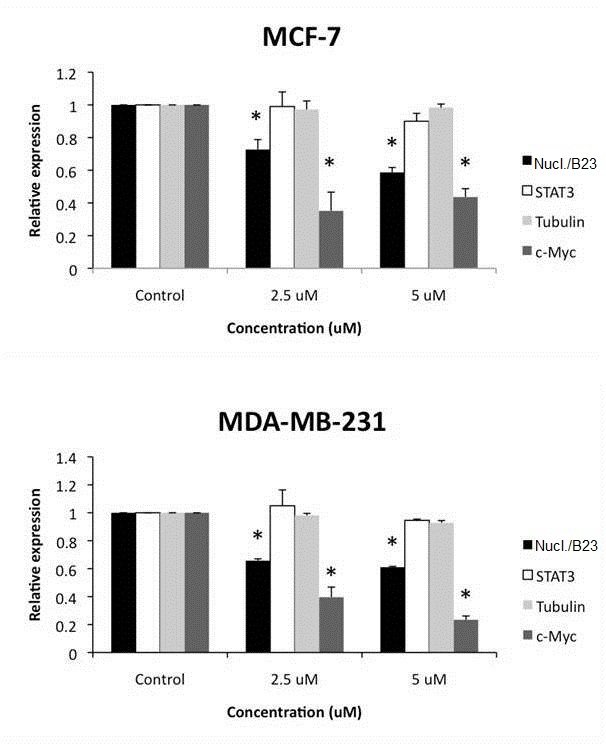


Figure Protein expression of nucleophosmin/B23 (Nucl./B23), STAT3, tubulin, and c-Myc were determined by western blot analysis. MCF-7 and MDA-MB-231 were treated without or with cucurbitacin B for 48 hrs. After incubation, total proteins were extracted and performed western blotting to analyze the expression levels of nucleophosmin, STAT3, tubulin, and c-Myc gene. This bar graph represents the densitometric analyses of expression of nucleophosmin/B23 (Nucl./B23), STAT3, tubulin, and c-Myc relative to the untreated control. * *P* < 0.05 (treated *vs* untreated control)


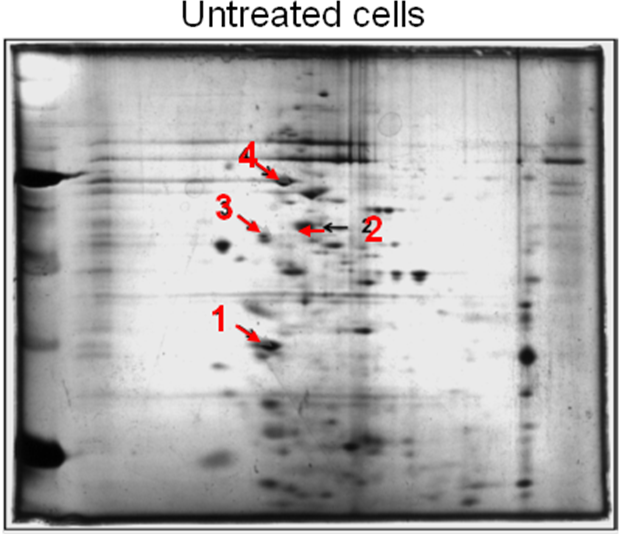


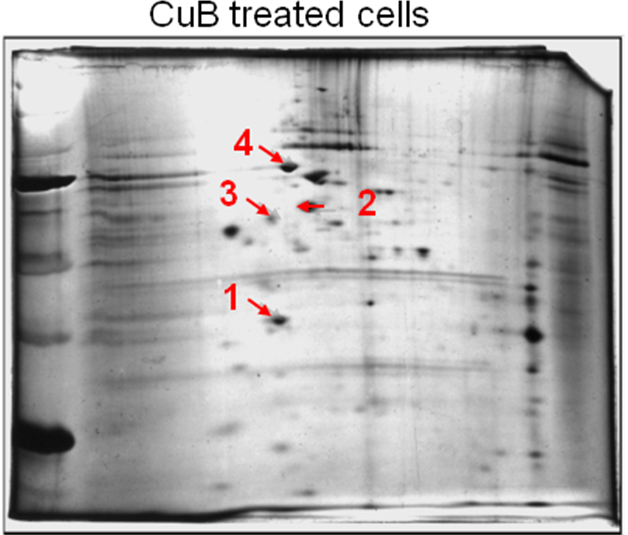


**Figure** 2D-PAGE electrophoresis. MCF-7 cells were collected after cucurbitacin B treatment for 48 hrs. Whole cells were lysed in RIPA buffer and protein was isolated. 100 µg protein samples were loaded on 7 cm IPG strip. The labels show the peptide spot IDs which related to the identifications listed in Table 1 A, 2D-PAGE from untreated cells. B, 2D-PAGE from cucurbitacin B treated cells (5 μM, 48 hrs)
